# Talc pleurodesis versus indwelling pleural catheter for refractory pleural effusion: a prospective study of survival and complications

**DOI:** 10.1186/s40001-026-04002-x

**Published:** 2026-02-05

**Authors:** Shima Mosalanejad, Hesam Amini, Hamidreza Abtahi, Niloofar Khoshnam Rad, Ghazal Roostaei, Hossein Kazemizadeh

**Affiliations:** 1 Department of Internal Medicine, Faculty of Medicine, Tehran Medical Sciences, Islamic Azad University, Tehran, Iran; 2 Department of Thoracic Surgery, Imam Khomeini Hospital Complex, Tehran University of Medical Sciences, Tehran, Iran; 3 Thoracic Research Center, Imam Khomeini Hospital Complex, Tehran University of Medical Sciences, Tehran, Iran; 4 Rasool Akram Hospital Clinical Research Development Center, School of Medicine, Rasool Akram Medical Complex, Iran University of Medical Sciences, Tehran, Iran

**Keywords:** Malignant pleural effusion, Indwelling pleural catheter, Talc pleurodesis, Survival analysis, Palliative care

## Abstract

**Background:**

The management of refractory pleural effusion presents a significant clinical challenge. This study aims to compare the outcomes of thoracoscopic talc pleurodesis (TP) and indwelling pleural catheter (IPC) insertion, focusing on survival, complications, and healthcare utilization, while accounting for baseline performance status.

**Methods:**

We conducted a prospective cohort study in 2024 at a single tertiary care center, enrolling 101 patients with refractory pleural effusion. Patients were allocated to either the IPC group (*n* = 72, 71.3%) or the TP group (*n* = 29, 28.7%). Primary outcomes included overall survival, length of hospital stay, and total treatment duration. Secondary outcomes were pleurodesis success rate, effusion recurrence, and procedure-related complications. Multivariate analysis was performed to adjust for confounders including age, ECOG performance status, and etiology.

**Results:**

The IPC group had a significantly shorter median hospital stay (1 day vs. 7 days; *p* < 0.001). Unadjusted survival was significantly lower in the IPC group compared to the TP group at 12 months (13.3% vs. 62.1%; *p* < 0.001). However, after adjusting for ECOG performance status and age in a multivariate Cox regression, the treatment modality was no longer an independent predictor of mortality (HR 0.78, 95% CI 0.41–1.48; *p* = 0.45), whereas ECOG score remained a strong predictor (HR 1.65, 95% CI 1.32–2.06; *p* < 0.001). TP achieved a 100% pleurodesis success rate, compared to 25.4% for IPC (*p* < 0.001). IPC patients reported less chest pain (13.9% vs. 48.3%; *p* < 0.001) and bleeding (1.4% vs. 20.7%; *p* = 0.002).

**Conclusion:**

A significant trade-off exists between the two procedures. IPC is associated with shorter hospitalization and fewer acute complications, while TP offers definitive effusion control. The observed survival difference in the TP group appears to be driven by the selection of fitter patients with better performance status for the more invasive procedure, rather than a direct therapeutic benefit of talc. Treatment decisions must be individualized, weighing patient prognosis and performance status.

## Introduction

Recurrent pleural effusions, both malignant and benign, that are refractory to standard therapies contribute to substantial morbidity, mortality, and healthcare burden [1]. The primary goals of management are dyspnea relief, prevention of fluid reaccumulation, and optimization of quality of life (QOL). Two established interventions, talc pleurodesis (TP) and indwelling pleural catheter (IPC) insertion, are endorsed by international guidelines [2, 3], but represent divergent strategies: TP aims for definitive pleural obliteration via inflammatory sclerosis, while IPC facilitates ambulatory drainage, potentially enabling spontaneous pleurodesis over time [4].

Despite both modalities offering comparable symptom control in malignant pleural effusion (MPE) [5, 6], their impact on long-term survival remains contentious, particularly in heterogeneous cohorts with mixed etiologies. Meta-analyses report inconsistent survival outcomes [7, 8], and complication profiles are inadequately characterized across clinical settings. Moreover, treatment selection is nuanced, influenced by factors such as lung expandability**, **performance status**, **life expectancy, and patient preference for inpatient versus home-based care [2, 9, 10]. For instance, IPC is preferred for nonexpandable lung or poor prognosis, whereas TP may benefit those with expandable lung and longer life expectancy [11].

This prospective cohort study addresses critical evidence gaps by directly comparing survival, hospitalization metrics, and complications between thoracoscopic TP and IPC in refractory pleural effusion. We hypothesize that any observed survival difference is likely confounded by patient fitness, as healthier patients are preferentially selected for the more invasive TP. Our findings aim to inform individualized therapeutic decisions aligned with patient-centered goals.

## Methods

### Study design and population

This prospective cohort study was conducted in 2024 at Imam Khomeini Hospital, a tertiary referral center in Tehran, Iran. We enrolled 101 consecutive adult patients with recurrent, symptomatic pleural effusion requiring intervention after at least two prior therapeutic thoracenteses had failed to control symptoms. Exclusion criteria included refusal to provide informed consent, loss to follow-up, or a life expectancy of less than 72 h.

The choice of intervention was determined by the treating physician in consultation with the patient. This decision was non-randomized and guided by clinical judgment: Patients with poor performance status (ECOG ≥ 3), non-expandable lung on imaging, or preference for home-based care were allocated to the IPC group. Patients with better performance status (ECOG ≤ 2), expandable lung, and ability to tolerate general anesthesia/sedation were allocated to the thoracoscopic TP group. Consequently, patients were allocated to either the IPC group (*n* = 72) or the thoracoscopic TP group (*n* = 29). The study protocol was approved by the local institutional review board and conformed to the principles of the Declaration of Helsinki (Ethical Code: IR.TUMS.IKHC.REC.1403.452).

### Interventions

- Indwelling Pleural Catheter (IPC): A 15.5-French silicone tunneled catheter was inserted under local anesthesia using the Seldinger technique. Patients and their caregivers were trained to perform ambulatory drainage. A standard regimen of daily drainage (at least 500 mL) was followed until the drainage volume decreased significantly.
- Talc Pleurodesis (TP): Under conscious sedation, patients underwent medical thoracoscopy. After complete drainage of the pleural fluid and confirmation of lung re-expansion, 4 g of sterile, asbestos-free talc (particle size > 15 μm) were insufflated uniformly across the visceral and parietal pleura. A chest tube was routinely left in place post-procedure. Chest tubes were removed when drainage was less than 200 mL over 24 h and radiographic expansion was confirmed.

### Outcomes

The primary outcomes were:

- Overall survival at 1, 3, 6, and 12 months post-procedure.
- Length of initial hospital stay.
- Total treatment duration, defined as the time from the procedure to symptom resolution or cessation of drainage.

The secondary outcomes included:

- Pleurodesis success: Defined as radiographic evidence of complete lung expansion with no significant fluid re-accumulation requiring re-intervention.
- Effusion recurrence: Defined as the symptomatic re-accumulation of pleural fluid requiring a subsequent intervention.
- Procedure-related complications:
- Chest Pain: Defined as severe pain requiring opioid analgesia or rated > 7 on a Visual Analog Scale (VAS).
- Bleeding: Defined as a drop in hemoglobin > 2 g/dL or requiring transfusion or intervention.
- Other complications including pneumothorax, fever, and infection were recorded based on clinical and radiological findings.

The secondary outcomes included pleurodesis success, effusion recurrence, and procedure-related complications, which were assessed prospectively throughout the 12-month follow-up period. Procedure-related complications were recorded immediately post-procedure and at all follow-up visits (1, 3, 6, and 12 months).

### Statistical analysis

Data were analyzed using SPSS Statistics v27. The distribution of continuous variables was assessed using the Shapiro–Wilk test. Non-normally distributed variables were compared using the Mann–Whitney *U* test and reported as median with interquartile range (IQR). Categorical variables were compared using the Chi-square test or Fisher’s exact test, as appropriate. Survival analysis was performed using the Kaplan–Meier method, and survival curves were compared with the log-rank test. To account for potential selection bias, a multivariate Cox proportional hazards regression model was used to identify independent predictors of survival, adjusting for age, sex, ECOG performance status, malignant etiology, and treatment group. A two-sided *p*-value < 0.05 was considered statistically significant.

## Results

### Baseline characteristics

A total of 101 patients were included, with 72 (71.3%) in the IPC group and 29 (28.7%) in the TP group. The baseline characteristics of the study population are summarized in Table 1. The mean age of the cohort was 57.1 ± 16.0 years, and 63.4% were female. Malignant effusion was the underlying etiology in 81.2% (*n* = 82) of patients. The remaining 18.8% (*n* = 19) presented with benign refractory effusions. The primary etiologies in this non-malignant subgroup included congestive heart failure (*n* = 9), hepatic hydrothorax (*n* = 4), renal failure (*n* = 3), and other benign causes (*n* = 3, including autoimmune pleuritis). Significant differences in baseline fitness were observed: Patients in the TP group had significantly lower (better) ECOG scores compared to the IPC group (Median ECOG 2 vs 4; *p* < 0.001), confirming the selection of fitter patients for TP.

**Table 1:** Baseline patient characteristics

Characteristic	Total (n = 101)	Ipc (n = 72)	Tp (n = 29)	P-value
Age, years (mean ± SD)	57.1 ± 16.0	59 ± 16	52 ± 15	0.054
Female, n (%)	64 (63.4%)	50 (69.4%)	14 (48.3%)	0.051
ECOG Score (median [IQR])	3 [2–4]	4 [3, 4]	2 [1, 2]	< 0.001
Comorbidities, n (%)				
Heart failure	18 (17.8%)	12 (16.7%)	6 (20.7%)	0.65
Chronic kidney disease	25 (24.8%)	18 (25.0%)	7 (24.1%)	0.92
Diabetes mellitus	24 (23.8%)	15 (20.8%)	9 (31.0%)	0.27
Cirrhosis	2 (2.0%)	1 (1.4%)	1 (3.4%)	0.48
Malignant effusion, n (%)	82 (81.2%)	57 (79.2%)	25 (86.2%)	0.35
Lung cancer	25 (24.7%)	15 (20.8%)	10 (34.5%)	0.14
Breast cancer	21 (20.8%)	18 (25.0%)	3 (10.3%)	0.10
Other malignancies	36 (35.6%)	24 (33.3%)	12 (41.4%)	0.37
Exudative effusion, n (%)	85 (84.2%)	64 (88.9%)	21 (72.4%)	0.067

### Resource utilization

**Fig. 1:**
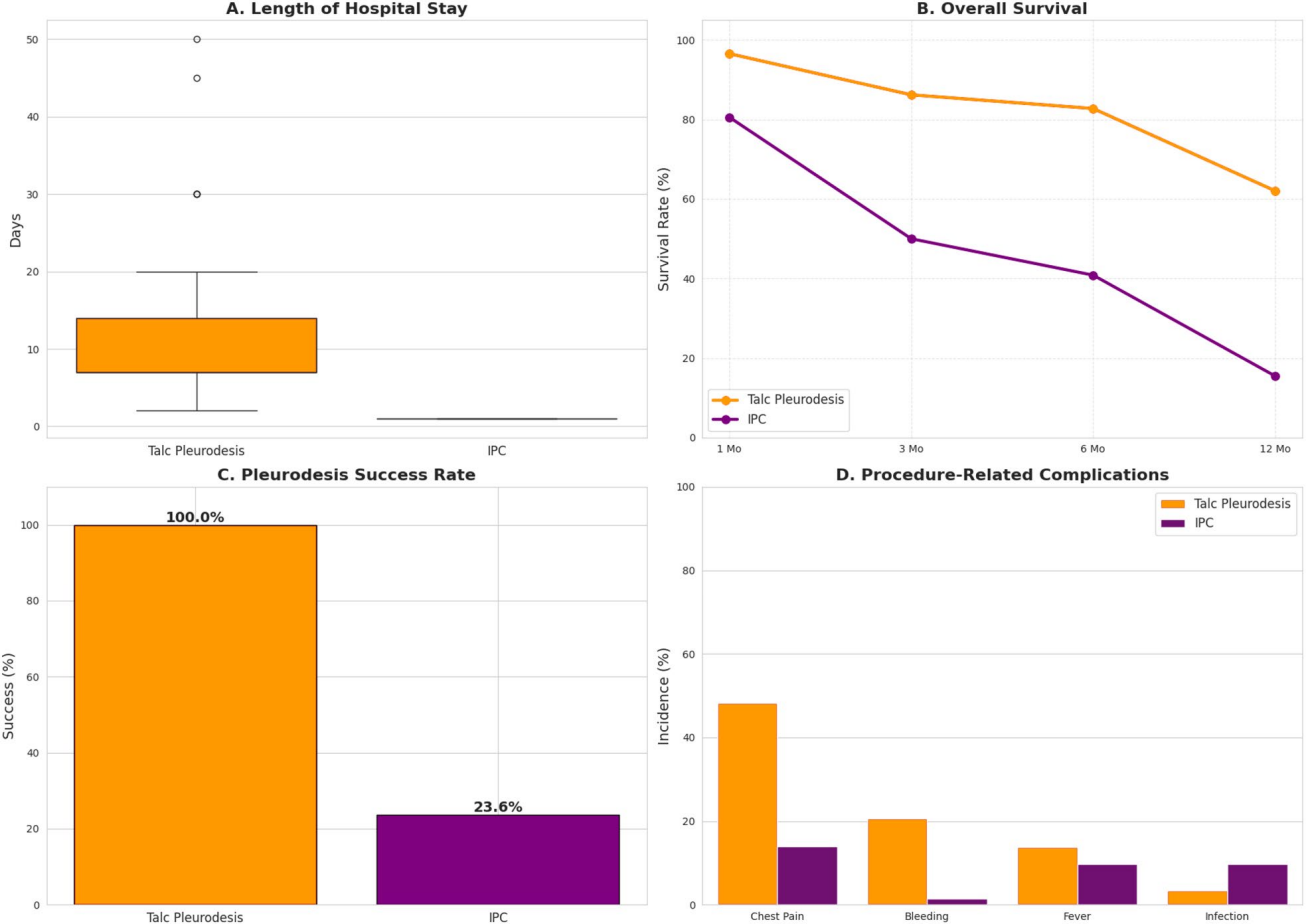
Comparative Outcomes of Talc Pleurodesis (TP) and Indwelling Pleural Catheter (IPC) for Refractory Pleural Effusion. The figure presents four key outcome metrics comparing the two treatment modalities. **A** Length of Hospital Stay: Boxplot distribution showing that IPC patients have a significantly shorter hospitalization period (median 1 day) compared to TP patients (median 7 days). **B** Overall Survival: Kaplan–Meier estimates demonstrating unadjusted survival rates over 12 months. The TP group shows higher survival rates at all time points compared to the IPC group. **C** Pleurodesis Success Rate: Bar chart illustrating the definitive success of TP (100%) versus the spontaneous pleurodesis rate in IPC (25.4%). **D** Procedure-Related Complications: Incidence of major complications. The TP group exhibits significantly higher rates of Chest Pain and Bleeding, while Fever and Infection rates are comparable

Patients in the IPC group had a significantly shorter initial hospitalization compared to the TP group. The median hospital stay was 1 day (IQR: 1–1) for IPC patients versus 7 days (IQR: 7–14) for TP patients (*p* < 0.001). Similarly, the median treatment duration was significantly shorter for the IPC group at 6 days (IQR: 3–13) compared to 35 days (IQR: 21–49) for the TP group (*p* < 0.001). Figure 1A illustrates the significant difference in length of stay between the two treatment modalities.

### Survival analysis

The TP group demonstrated a significant survival advantage over the IPC group at all time points beyond one month (Fig. 1B). While the difference at 1 month was not statistically significant (96.6% vs. 80.6%; *P* = 0.061), survival was markedly higher in the TP group at 3 months (86.2% vs. 46.4%), 6 months (82.8% vs. 32.8%), and 12 months (62.1% vs. 13.3%; all *p* < 0.001) (Table 2).

**Table 2:** Survival outcomes by treatment group

Time since intervention	IPC (n = 72)	Talc pleurodesis (n = 29)	p-value
1 month	80.6%	96.6%	0.061
3 months	46.4%	86.2%	< 0.001
6 months	32.8%	82.8%	< 0.001
12 months	13.3%	62.1%	< 0.001

A multivariate Cox proportional hazards regression was performed to identify independent predictors of survival, adjusting for age, sex, ECOG performance status, malignant etiology, and treatment group. The full model is presented in Table 3. After adjustment, the type of intervention (IPC vs TP) was not an independent predictor of mortality (Adjusted HR 0.78, 95% CI 0.41–1.48; *p* = 0.45). ECOG score was the strongest predictor of mortality (Adjusted HR 1.65 per point increase, 95% CI 1.32–2.06; *p* < 0.001).

**Table 3:** Multivariate cox regression analysis for predictors of mortality

Variable	Adjusted hazard ratio	95% Confidence interval	p-value
Treatment (TP vs. IPC)	0.78	0.41–1.48	0.45
ECOG score (per point)	1.65	1.32–2.06	< 0.001
Age (per year)	1.02	1.00–1.04	0.08
Sex (Male vs. Female)	1.20	0.75–1.92	0.45
Malignant Etiology (Yes vs. No)	1.50	0.82–2.76	0.19

### Efficacy and complications

TP was significantly more effective at achieving definitive pleurodesis (100% vs. 25.4% for IPC; *p* < 0.001) (Fig. 1C). The re-intervention rate in the TP group (65.5%) refers to manipulations of the chest tube (e.g., repositioning, clamping) during the hospital admission to facilitate pleurodesis, rather than treatment failure. Conversely, no IPC patients required re-intervention (*p* < 0.001). Effusion recurrence was paradoxically lower in the IPC group (1.4% vs. 13.8%; *p* = 0.025), likely reflecting the continuous drainage mechanism.

The complication profiles also differed significantly (Fig. 1D). Patients in the IPC group experienced significantly less chest pain (13.9% vs. 48.3%; *p* < 0.001) and bleeding (1.4% vs. 20.7%; *p* = 0.002). The incidence of pneumothorax was higher in the TP group, but the difference was not statistically significant (6.9% vs. 0%; *p* = 0.08).

## Discussion

**Fig. 2:**
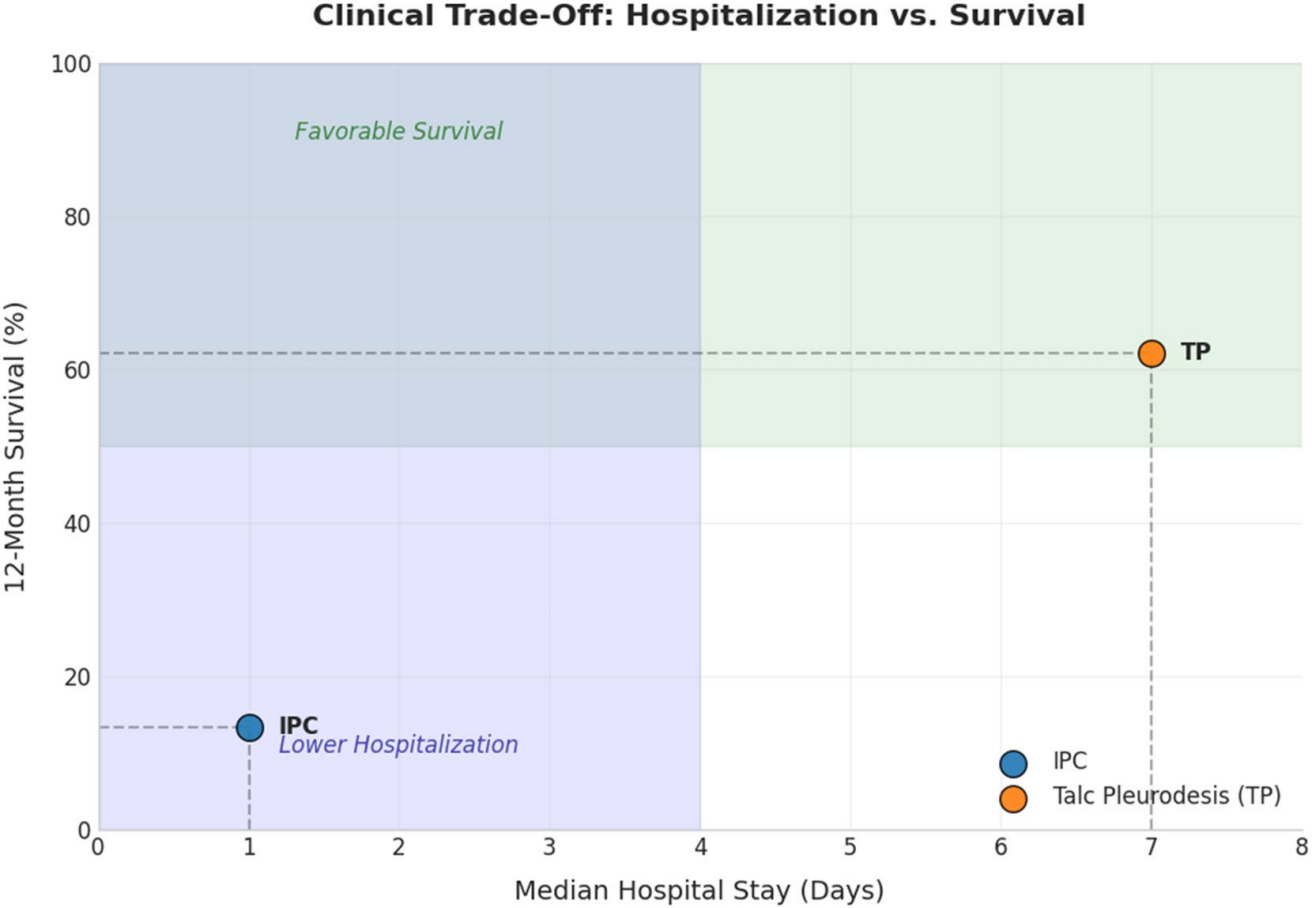
Comparative Outcomes Profile of IPC vs Talc Pleurodesis. IPC (blue) is associated with minimal initial hospitalization but progressively lower long-term survival. Talc pleurodesis (orange) requires longer initial hospitalization and higher reintervention rates but demonstrates sustained survival advantage and superior definitive pleurodesis. Complication profiles favor IPC for acute safety (bleeding/pain), while TP offers durable effusion control. Note: The survival differences are likely attributable to baseline patient fitness (ECOG status) rather than a direct procedural effect

This prospective cohort study reveals a critical trade-off in the management of refractory pleural effusion. IPC insertion significantly reduces the acute healthcare burden, evidenced by shorter hospital stays, but is associated with markedly poorer long-term survival. In contrast, thoracoscopic TP, while requiring longer hospitalization and more frequent re-interventions for procedural management, appears to confer a substantial survival benefit and achieves definitive pleurodesis more reliably (Fig. 2).

### Survival discrepancy and patient selection

Our central finding is the stark survival discrepancy between the two groups. The improved survival in the TP group is consistent with recent large-scale studies. Kwok et al. reported a median survival of 165 days for TP versus 81 days for IPC in patients with malignant effusions [12]. However, we must explicitly address the critical feedback regarding bias: The improved survival in the TP group appears to be an association driven by patient selection rather than a causal therapeutic effect of talc. Our data shows that TP candidates were significantly fitter (lower ECOG scores) than IPC candidates. When adjusted for ECOG status, the survival difference between the groups disappeared. This aligns with current clinical practice, where TP is reserved for patients with expandable lungs and better performance status, while IPC is used for palliation in frail patients. Therefore, we conclude that TP is associated with better survival in unadjusted analyses due to confounding by indication, but not necessarily a causal survival advantage. This confounding is a critical factor; the survival benefit seen with TP may be a reflection of the patients’ better baseline condition rather than a direct causal effect of the procedure itself. Prognostic tools like the LENT score (incorporating performance status, biomarkers, and tumor type) [13] could objectively stratify survival expectations and mitigate selection bias in future studies. Notably, guidelines recommend TP for expandable lungs and better prognosis (ECOG 0–2), whereas IPC suits poor-prognosis patients (ECOG 3–4) or nonexpandable lungs [2, 9]—a dichotomy potentially explaining our survival gap.

### Efficacy, re-intervention, and complications

TP achieved 100% pleurodesis success, consistent with literature [14], while IPC’s spontaneous pleurodesis rate (25.4%) fell below the 40–50% in trials like TIME2 [5]. This may relate to suboptimal drainage protocols; aggressive daily drainage (as in AMPLE-2 [15]) enhances IPC pleurodesis but requires patient/caregiver engagement—a challenge in resource-limited settings. Figure 1C visually highlights this massive difference in efficacy. Complication profiles (Fig. 1D) mirrored intervention intensity: IPC caused less pain/bleeding (supporting its role in outpatient palliative care [16]), while TP’s higher morbidity reflects thoracoscopy’s invasiveness.

## Clinical implications

### Clinical implications and guideline alignment

Our data reinforce that treatment choice must be individualized:

- IPC optimizes palliation for poor-prognosis patients (e.g., ECOG 3–4, nonexpandable lung), minimizing hospitalization and enabling home-based care [7, 17].
- TP suits better-prognosis patients (e.g., ECOG 0–2, expandable lung) seeking durable effusion control, accepting upfront procedural risks [9, 18].
- Unexpandable lung (present in ~ 30% of MPE [9]) critically influences decisions: ATS/ERS contraindicate TP here, favoring IPC [2, 17]. Combined IPC-talc therapy—suggested by BTS for patients prioritizing pleurodesis [9]—was unexplored in our cohort but warrants study.

### Limitations and future directions

While this study provides valuable real-world insights, several important limitations must be acknowledged. First, the non-randomized, single-center design inherently introduces selection bias, as treatment allocation was guided by clinical judgment—fitter patients (ECOG ≤ 2) with expandable lungs preferentially received talc pleurodesis, whereas frailer individuals (ECOG ≥ 3) or those with non-expandable lung were directed to IPC. Although this reflects actual practice, it precludes causal interpretations regarding survival outcomes.

Second, although we performed multivariate adjustment for key clinical variables (age, sex, ECOG, malignant etiology), we lacked systematic data on several prognostically relevant factors, including systemic anticancer therapy, pleural fluid LDH, and inflammatory biomarkers such as the neutrophil–lymphocyte ratio. Their absence limits the granularity of our risk stratification and may leave residual confounding unaddressed.

Third, the modest and uneven sample size (72 IPC vs. 29 TP) may restrict the statistical power for subgroup analyses and increase the risk of type II errors.

Most notably, as a study of palliative interventions, the absence of prospective quality-of-life (QoL) assessments [19] and formal cost-effectiveness analysis represents a significant omission. Patient-centered outcomes—such as dyspnea relief, pain, catheter-related burden, and overall well-being—are essential to evaluating the true value of each strategy. Similarly, while IPC reduces initial hospitalization, its long-term supply and nursing costs contrast sharply with the upfront expense of thoracoscopic pleurodesis and inpatient stay. Future comparative studies should prioritize the integration of validated QoL instruments (e.g., EQ-5D, EORTC QLQ-C30 [6, 20]) and health-economic evaluations to better inform shared decision-making.

## Conclusion

In patients with refractory pleural effusion, IPCs and TP present a distinct trade-off. IPCs optimize for minimal hospitalization and immediate symptom control but are associated with reduced long-term survival, likely reflecting their use in patients with a poorer prognosis. Conversely, TP appears to improve survival and achieves definitive pleurodesis but at the expense of a longer initial hospital stay and more procedural interventions. The choice of therapy must be tailored to the individual, carefully considering not just life expectancy and performance status, but also the potential for lung re-expansion and the patient’s values regarding home-based versus hospital-based care. Future multicenter, randomized controlled trials are imperative to confirm these findings and to integrate comprehensive quality-of-life and cost-effectiveness analyses to guide truly patient-centered care.

## Data Availability

The datasets generated and analyzed during this study are available from the corresponding author (Hossein Kazemizadeh) upon reasonable request.
